# Иммунометаболические нарушения при сахарном диабете 2-го типа, опосредованные активацией инфламмасомы NLRP3, и способы их фармакологической коррекции

**DOI:** 10.14341/probl13590

**Published:** 2026-01-18

**Authors:** Н. И. Чепляева, Д. А. Бабков, А. В. Лукьянов, Р. Д. Данилов, А. А. Спасов

**Affiliations:** Волгоградский государственные медицинский университетРоссия; Volgograd State Medical UniversityRussian Federation

**Keywords:** сахарный диабет 2 типа, инфламмасома, ингибитор NLRP3, интерлейкин-1β, воспаление, type 2 diabetes mellitus, inflammasome, NLRP3 inhibitor, interleukin-1β, inflammation

## Abstract

Согласно последним исследованиям, хроническое системное воспаление, опосредованное активацией инфламмасомы NOD-подобного рецепторного белка 3 (NLRP3), является одним из ключевых факторов в патофизиологии сахарного диабета (СД) 2-го типа. Основные особенности активации сигнальных каскадов и регуляторных механизмов инфламмасомы NLRP3 при СД 2-го типа (СД2) связаны с тем, что глюкоза, насыщенные жирные кислоты, липотоксичные церамиды, окисленные ЛПНП и холестерин выступают в качестве основных молекулярных паттернов, ассоциированных с повреждением активирующих инфламмасому и запускающих каскад сигнальных механизмов, приводящих к выработке ИЛ-1β и провоспалительных цитокинов. Ряд противодиабетических препаратов не только эффективно контролирует уровень глюкозы, но и корректирует иммунометаболические нарушения, связанные с активацией инфламмасомы NLRP3. Учитывая роль интерлейкина-1β (ИЛ-1β) в воспалении, связанном с СД2, препараты анти-ИЛ-1 терапии, такие как анакинра, канакинумаб, гевокизумаб, исследуются как в экспериментальных моделях СД, так и в клинических испытаниях. Однако применение данной группы ограничивается увеличенным риском инфекционных заболеваний. Среди ингибиторов активации инфламмасомы NLRP3 наиболее исследованными являются MCC950, OLT1177, CY-09, но ни одно из соединений данной группы в настоящее время не применяется в клинической практике. Целью настоящего обзора является оценка роли инфламмасомы NLRP3 в патогенезе СД2, а также потенциала ингибиторов инфламмасомного пути как перспективных средств его терапии.

## Abstract (en)

According to recent studies, chronic systemic inflammation mediated by activation of the inflammasome NOD-like receptor protein 3 (NLRP3) is a key factor in the pathophysiology of type 2 diabetes mellitus (DM). The main features of the activation of signalling cascades and regulatory mechanisms of the NLRP3 inflammasome in type 2 DM are related to the fact that glucose, saturated fatty acids, lipotoxic ceramides, oxidised LDL and cholesterol act as the main molecular patterns associated with damage, activating the inflammasome and triggering a cascade of signalling mechanisms leading to the production of IL-1β and pro-inflammatory cytokines. A number of antidiabetic drugs not only effectively control glucose levels, but also correct immunometabolic disorders associated with NLRP3 inflammasome activation. Given the role of interleukin-1β (IL-1β) in the inflammation associated with type 2 DM, anti-IL-1 therapies such as anakinra, canakinumab and gevokizumab are being investigated in both experimental models of DM and clinical trials. However, the use of this group is limited by the increased risk of infection. Among the inhibitors of NLRP3 inflammasome activation, MCC950, OLT1177, CY-09 are the most studied, but none of the compounds in this group are currently used in clinical practice. The aim of this review is to assess the role of the NLRP3 inflammasome in the pathogenesis of type 2 diabetes, as well as the potential of inflammasome pathway inhibitors as promising therapeutic agents.

Сахарный диабет (СД) является мультифакторным заболеванием с растущей распространенностью. По данным Международной федерации диабета, количество пациентов с СД в возрасте 20–79 лет в мире достигло 537 млн, что опережает ранее прогнозируемые темпы прироста на 10–12 лет, а к 2045 г. ожидается практически двукратное увеличение до 783 млн человек [1]. Основными составляющими, играющими ведущую роль в патогенезе СД2, является прогрессирующее снижение массы и функционального резерва β-клеток и резистентность периферических тканей к инсулину, ключевым фактором в развитии которых играет хроническое системное воспаление, опосредованное активацией инфламмасомы NLRP3. В последние годы достигнут значительный прогресс в исследовании механизмов активации инфламмасомы и их роли в патогенезе различных заболеваний, что способствует разработке терапевтических подходов, направленных на снижение системного хронического воспаления, опосредованного инфламмасомой NLRP3 [2].

## ИММУНОМЕТАБОЛИЧЕСКИЕ СИГНАЛЬНЫЕ ПУТИ, РЕГУЛИРУЕМЫЕ ИНФЛАММАСОМОЙ NLRP3

Врожденный иммунитет обеспечивает быструю и консервативную защиту при повреждении клеток, вызванном патогенами, травмами и клеточным стрессом. Ключевую роль в восприятии провоспалительных сигналов и запуске врожденного иммунного ответа выполняют крупные белковые комплексы, называемые инфламмасомами [2][3].

Активация инфламмасом запускает воспалительные реакции, которые регулируют широкий спектр биологических процессов, включая транскрипционные пути, опосредованные ядерным фактором каппа B (nuclear factor kappa B, NF-κB) и митоген-активируемой протеинкиназой (mitogen-activated protein kinase, МАРК), презентацию антигена, аутофагию, эмбриональное развитие и сборку цитоплазматического сигнального трансдукционного комплекса [4].

Инфламмасома состоит из цитозольного сенсорного комплекса NLR; апоптоз-ассоциированного спек-подобного белка, содержащего CARD домен (apoptosis-associated speck-like protein containing a CARD, ASC) на C-конце и пиринового домена (PYD) на N-конце и цистеиновой протеазы и прокаспазы-1, содержащей каспазу-1 и CARD (рис. 1) [2–4].

**Figure:**
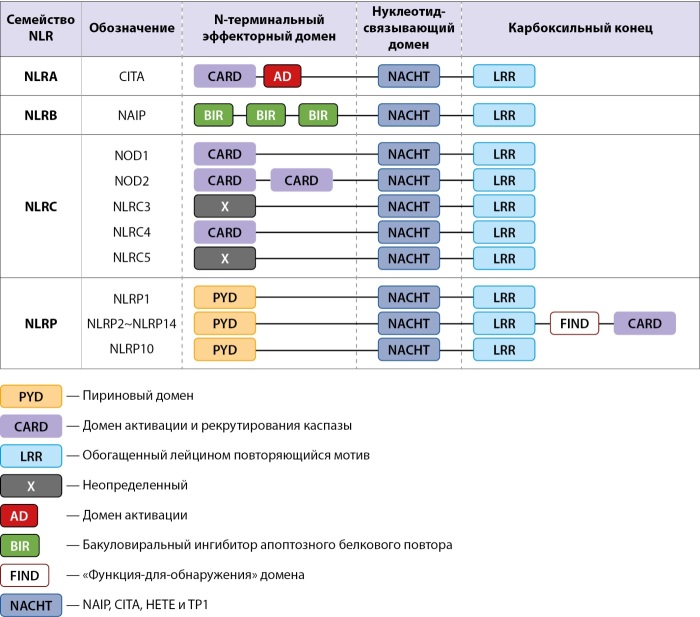
Рисунок 1. Семейство NLR человека.

Белки NLR являются цитозольными паттерн-распознающими рецепторами (pattern recognition receptors, PRR), которые распознают высококонсервативные структуры микроорганизмов, так называемые патоген-ассоциированные молекулярные паттерны (patogen-associated molecular patterns, PAMP), и молекулярные паттерны, ассоциированные с повреждением — DAMP (damage-associated molecular patterns). Среди всех PRR NLR — одно из многочисленных и разнообразных семейств с 22 идентифицированными рецепторами у человека. Структурно членов данного семейства объединяет сходная архитектура доменов [4]. Семейство NLR подразделяется на четыре подгруппы: NLRA, NLRB, NLRC и NLRP, в зависимости от природы N-концевого домена: NLRA и NLRC имеют домены кислотной трансактивации и рекрутирования каспаз (CARD), семейство NLRB обладает доменом, подобным бакуловирусному ингибирующему повтору (BIR), семейство NLRP содержит PYD [2][4]. Инфламмасомные комплексы обозначают по типу NRL белка, входящего в их состав. Таким образом, выделяют инфламмасомы NRLP1, NRLP3, NRLP6, NRLP7, NRLP12 и NRLC4 (рис. 1) [5].

Из всех типов инфламмасом наибольшее внимание исследователи уделяют NRLP3, уникальному представителю семейства рецепторов NRL, распознающему PAMP и DAMP и опосредует развитие стерильной воспалительной реакции при различных заболеваниях. Лигандами NRLP3 могут выступать бактериальные и вирусные PAMP, такие как ЛПС и нуклеиновые кислоты, порообразующие бактериальные токсины, такие как нигерицин и грамицидин, активные формы кислорода (АФК) и твердые частицы асбеста, кремния, кристаллы мочевой кислоты, холестерин, β-амилоид, кристаллы солей кальция [2][4][5][6].

NLR в NLRP3 включает N-концевой эффекторный домен, центральный нуклеотидсвязывающий домен (NACHT), который участвует в АТФ-зависимой олигомеризации и С-концевую область, состоящую из повторов, богатых лейцином (LLR). NACHT- и LLR-домены высококонсервативны во всех NLR, а N-концевой домен весьма вариабелен и определяет паттерн, с которых взаимодействует рецептор, и конечный эффект. Домен PYD играет роль в рекрутировании белка ASC после активации инфламмасомы NLRP3, домен NACHT функционирует как АТФаза, в которой мотив Walker A содержит сайт связывания АТФ, а мотив Walker B необходим для активности АТФазы с последующей олигомеризацией и функционированием NLRP3. Домен LRR, состоящий из 12 повторов, выполняет более сложные функции. LRR играет аутоингибиторную роль при формировании неактивной двухкольцевой структуры «клетки» («бочки») посредством взаимодействия «face-face» и «back-back», используя свои вогнутые и выпуклые стороны. Помимо этого, домен LRR может подвергаться нескольким посттрансляционным модификациям, таким как деубиквитинирование и фосфорилирование при восприятии PAMP и DAMP, играя роль в активации NLRP3 [4–6].

Попытки выяснить общий механизм, который связывает различные сигналы, привели к открытию трех различных путей регуляции активации NLRP3. К ним относятся канонический или классический путь, неканонический путь и, совсем недавно обнаруженный, альтернативный путь [3].

Активация NLRP3 по каноническому пути является двухэтапным процессом, который включает начальный этап праймирования (или прайминга), за которым следует последующий этап активации (рис. 2) [2–7].

**Figure:**
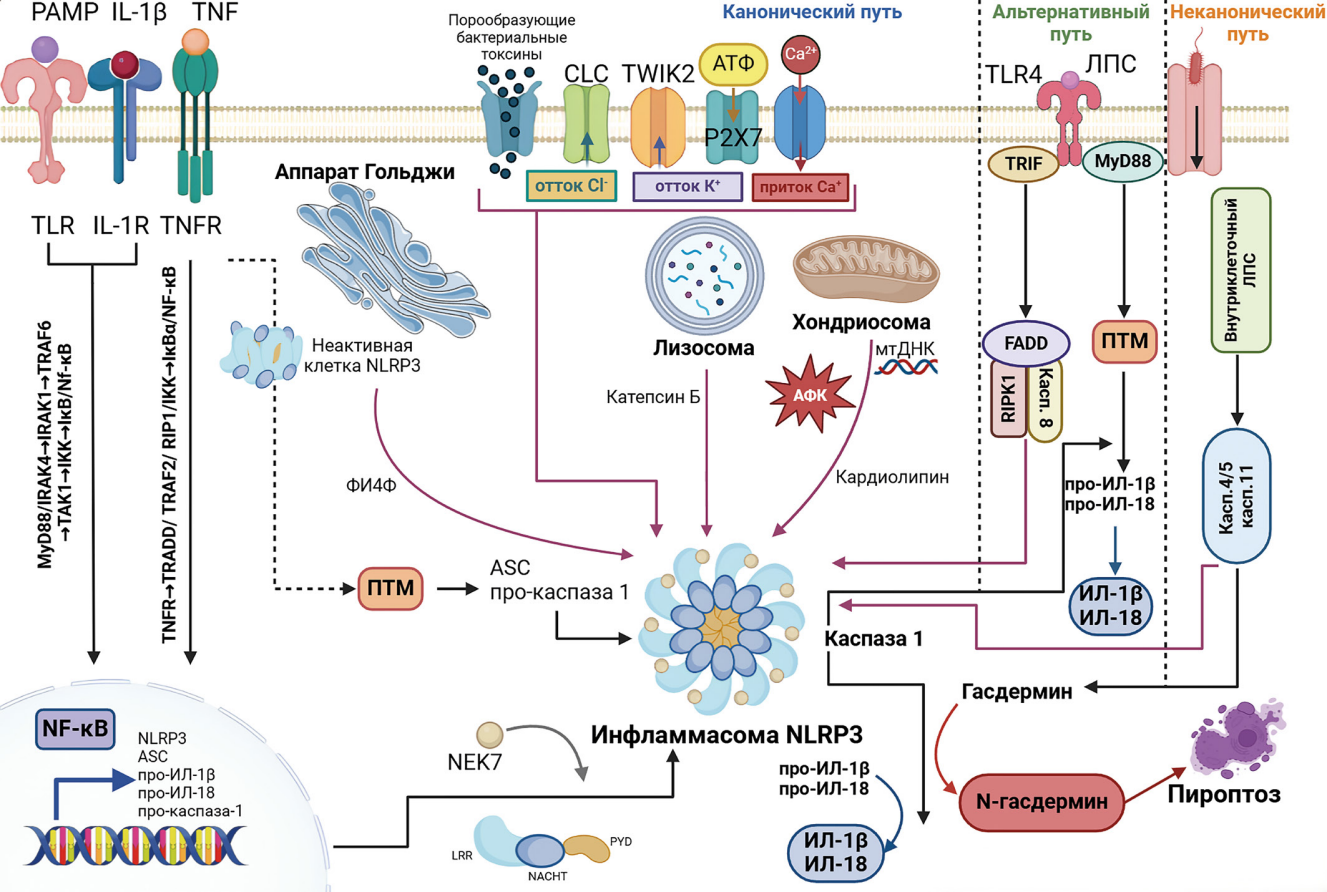
Рисунок 2. Клеточные пути активации инфламмасомы NLRP3.

Традиционно считалось, что праймирование в первую очередь зависит от транскрипционной регуляции, при этом стимуляция TLR приводит к повышению экспрессии про-ИЛ-1β, про-ИЛ-18 и самих белков инфламмасомы NLRP3 через NF-κB-зависимые сигнальные пути. Однако данные представления были оспорены, когда было доказано, что TLR-индуцированный прайминг NLRP3-инфламмасомы может осуществляться нетранскрипционно и не требует синтеза новых белковых молекул или повышения уровня NLRP3 [4].

На ранней фазе активация инфламмасомы не зависит от синтеза нового белка, а напрямую регулируется сигнализацией TLR через сигнальную молекулу киназу 1, ассоциированную с рецептором ИЛ-1 (interleukin-1 receptor-associated kinase 1, IRAK-1). IRAK-1-зависимый путь активации инфламмасомы NLRP3 имеет решающее значение для пироптоза и секреции воспалительных белков, предварительно синтезированных клеткой, что подтверждает прямую связь между сигнализацией TLR и активацией инфламмасомы NLRP3 и является критическим фактором транскрипционно независимого, инфламмасомозависимого раннего ответа на патоген [8].

Другая функция прайминга — индукция ПТМ NLPR3, таких как убиквитинирование, фосфорилирование и сумоилирование [9–11]. Необходимо отметить, что множество различных белков участвует в ПТМ компонентов инфламмасомы NLRP3, изменяя функцию, активность, внутриклеточное расположение и, следовательно, регулируя активацию инфламмасомы NLRP3. Регуляция инфламмасомы NLRP3 с помощью ПТМ открывает новые мишени для профилактики и терапии заболеваний, опосредованных NLRP3.

Второй этап представляет собой запуск ряда клеточных событий под влиянием PAMP или DAMP, приводящих непосредственно к активации инфламмасомы NLRP3. Так как широкий спектр агентов может выступать в качестве активатора, то маловероятно, что все агенты напрямую связываются с NLRP3 и активируют ее, поскольку они имеют различные структурные особенности, следовательно, NLRP3 может распознавать определенные медиаторы или вторичные сигналы [2][4].

В качестве сигналов, которые запускают второй этап активации инфламмасомы по каноническому пути активации, могут выступать изменение ионного гомеостаза (отток калия, мобилизация кальция), митохондриальная дисфункция, повреждение лизосом, стресс эндоплазматического ретикулума и дисперсия Гольджи [10][12].

Помимо классического пути, инфламмасома NLRP3 может быть активирована при непосредственном взаимодействии в цитозоле бактериального ЛПС с каспазой-11 при инфицировании грамотрицательными бактериями (рис. 3) [4].

**Figure:**
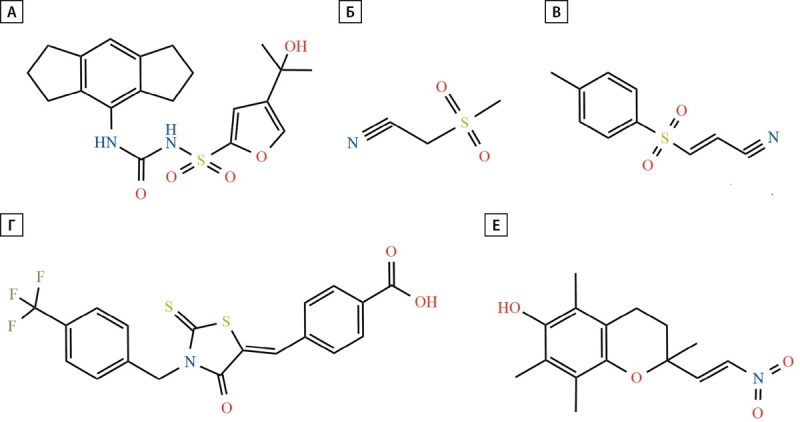
Рисунок 3. Структуры низкомолекулярных ингибиторов инфламмасомы NLRP3.

При избыточном содержании в свободной форме или в составе вакуолей ЛПС способен проникать во внутриклеточное пространство независимо от TLR4. При этом ЛПС из внутриклеточных грамотрицательных бактериальных патогенов, которые находятся внутри мембраны или фагосомы, при участии гуанилат-связывающих белков и интерферон-индуцированных ГТФаз попадают в цитозоль и запускают активацию каспазы-11 мыши и каспазы-4/5 человека [13]. GSDMD является субстратом активной каспазы-4/5/11, что приводит к образованию пор и пироптотической гибели клеток [6][13]. Активация каспазы-11, вызванная ЛПС, приводит к активации инфламмасомы NLRP3, вероятно, через механизмы, которые включают обработку паннексином-1 и отток калия. Так как сама каспаза-11 не обладает способностью расщеплять про-ИЛ-1β и про-ИЛ-18, активация инфламмасомы NLRP3 запускает расщепление каспазы-1 и приводит к секреции ИЛ-1β и ИЛ-18, характерных для канонического сигнального пути [13].

В дополнение к канонической и неканонической активации NLRP3, существует еще один клеточно- и видоспецифичный, так называемый альтернативный путь, по которому сигнал передается через TLR4 при связывании с ЛПС в моноцитах человека и свиньи, но отсутствует у мышей. Данный путь активации не зависит от АТФ-зависимого оттока калия. Сборка инфламмасомы происходит после активации TLR4 с помощью ЛПС, запускающего сигнальный каскад TRIF-RIPK1-FADD, который, в свою очередь, приводит к активации инфламмасомы NLRP3. Пироптоза не происходит, поэтому ИЛ-1β высвобождается постепенно, в отличие от реакции «все или ничего», характерной для канонической активации [4][6].

Активированная каспаза-1 в свою очередь расщепляет и активирует цитозольный проинтерлейкин (ИЛ)-1β и проинтерлейкин (ИЛ)-18, преобразуя те в зрелые биологически активные формы. Одновременно с расщеплением ИЛ-1β и ИЛ-18 расщепляется гасдермин D (GSDMD), что приводит к регулируемой форме воспалительной гибели клеток, известной как пироптоз [34]. После расщепления GSDMD N-концевой домен освобождается и может свободно олигомеризоваться и встраиваться в плазматическую мембрану, что приводит к образованию пор, потере осмотического гомеостаза, набуханию клетки и гибели. Следует отметить, что экспрессия N-концевого домена сама по себе достаточна для запуска пироптоза. N-концевой домен связывается с фосфолипидами, такими как кардиолипин, фосфатидилинозитол-4-фосфат и фосфатидилинозитол-4,5-бисфосфат, фосфатидилсерин, которые присутствуют на бактериях, митохондриях и в плазматической мембране. Связывание N-концевого домена GSDMD вызывает олигомеризацию протомеров, что приводит к образованию пор в мембране. Так как фосфолипиды находятся во внутреннем слое здоровых клеток, N-концевой GSDMD вызывает образование пор и лизис только изнутри [14].

## ИММУНОМЕТАБОЛИЧЕСКИЕ НАРУШЕНИЯ ПРИ САХАРНОМ ДИАБЕТЕ 2-ГО ТИПА, ОПОСРЕДОВАННЫЕ АКТИВАЦИЕЙ ИНФЛАММАСОМЫ NLRP3

Факторы врожденного иммунитета и инфламмасома NLRP3 играют важную роль в патогенезе СД и его осложнений [15–20]. Исследования Dror E. и соавт. демонстрируют, что прием пищи вызывает физиологическое повышение уровня ИЛ-1β, который секретируется перитонеальными макрофагами, что способствует секреции инсулина. Этот эффект зависит от бактериальных продуктов, которые стимулируют макрофаги вырабатывать больше про-ИЛ-1β, и от глюкозы, которая стимулирует созревание ИЛ-1β. Выработка ИЛ-1β макрофагами M1 типа и в меньшей степени M0 усиливается инсулином. Как инсулин, так и ИЛ-1β, регулируют утилизацию глюкозы, но ИЛ-1β преимущественно стимулирует поглощение глюкозы иммунными клетками. Однако гиперактивация данной системы ограничивается нормализацией гликемии. Так, снижение гликемии посредством ингибирования натрий-глюкозного котранспортера 2-го типа (SGLT2) или блокирования гликолиза с помощью 2-дезоксиглюкозы предотвращает постпрандиальную продукцию ИЛ-1β. Кроме того, исследователи показывают, что введение ИЛ-1β in vivo значительно усиливает секрецию инсулина в присутствии глюкозы. Физиологическое влияние постпрандиальной секреции ИЛ-1β на гомеостаз глюкозы не согласуется с данными о неблагоприятных эффектах цитокина на функцию и выживаемость β-клеток островков. Несмотря на то, что ИЛ-1β участвует в гибели β-клеток, в низких концентрациях или при кратковременном воздействии ИЛ-1β парадоксальным образом стимулирует пролиферацию β-клеток и снижает апоптоз, что свидетельствует о достаточно сложных регуляторных механизмах [17][21].

Данные свидетельствуют, что хроническая стимуляция секреции ИЛ-1β приводит к увеличению уровня инсулина, негативно влияя на метаболизм. Возможно, инсулин повышает иммунный статус макрофагов, стимулированных к захвату и метаболизму глюкозы, и экспрессию рецепторов инсулина на клетках макрофагов. Повышенный уровень инсулина на ранних стадиях СД2 может поддерживать макрофаги в активированном состоянии и, следовательно, может способствовать хроническому слабовыраженному воспалению, связанному с метаболическими заболеваниями [3][21].

Некоторые исследования подтверждают, что инфламмасома NLRP3 может активироваться в ответ на хроническую гипергликемию; однако мало что известно о влиянии острых сдвигов глюкозы на активацию инфламмасомы NLRP3. Согласно Lee J.Y. и соавт., увеличение в среде концентраций глюкозы от 5,5 до 25 мМ или снижение концентрации глюкозы от 25 до 5,5 мМ повышает активацию инфламмасомы NLRP3, генерацию АФК и экспрессию фосфорилированного p38 MAPK, JNK и NF-κB по сравнению с постоянной нормогликемией или гипергликемией [22].

Гипергликемия, гиперлипидемия и воспаление являются мощными факторами, способствующими выработке АФК в β-клетках. В дополнение к окислительному стрессу, напрямую вызванному гипергликемией, гиперлипидемией и воспалением, митохондриальная дисфункция и вторичный по отношению к этим состояниям стресс эндоплазматического ретикулума еще больше увеличивает выработку АФК. Окислительный стресс изменяет основные пути, важные для функционирования и выживания β-клеток, и активирует AMP-активируемую протеинкиназу (AMPK), c-Jun N-терминальную киназу (JNK) и ингибирует мишень рапамицина млекопитающих (mTOR). Инактивация mTOR1, опосредованная активацией AMPK, имеет ряд отрицательных эффектов, одним из которых является повышение экспрессии белка, взаимодействующего с тиоредоксином (thioredoxin interacting protein TXNIP), и перемещение его в митохондрии в условиях окислительного стресса. TXNIP является повсеместно экспрессируемым белком, который влияет на клеточный окислительно-восстановительный баланс посредством негативной регуляции антиоксидантных систем тиоредоксина. В покоящихся клетках TXNIP связан с тиоредоксином и недоступен для взаимодействия с NLRP3. При увеличении АФК TXNIP высвобождается из окисленного тиоредоксина и связывается NLRP3. В дополнение к оси АФК-TXNIP снижение цитоплазматического калия имеет важное значение для активации инфламмасомы NLRP3 и последующего высвобождения ИЛ-1β. Индуцированная TXNIP сборка инфламмасомы NLRP3 приводит к активации прокаспазы-1, которая затем вызывает гибель клеток посредством образования микропор в плазматической мембране и секрецию ИЛ-1β [15][17][23].

Кроме того, СД2 часто сопровождается избыточной секрецией островкового амилоидного полипептида (IAPP), большое количество которого агрегирует в преципитаты амилоида IAPP, которые откладываются в островковых клетках Лангерганса. IAPP представляет собой пептидный гормон, секретируемый β-клетками вместе с инсулином. Ранее было высказано предположение, что активация инфламмасомы и гибель панкреатических β-клеток, опосредованная IAPP, зависит от продукции АФК. По данным Morikawa S. и соавт., IAPP напрямую взаимодействует с доменом LRR NLRP3 и активирует инфламмасому. Таким образом, прямая активация инфламмасомы NLRP3 в β-клетках островков Лангерганса IAPP может способствовать воспалению и гибели β-клеток при СД2 в дополнение к механизму, опосредованному АФК [24].

Накапливающиеся доказательства подчеркивают центральную роль инфламмасомы NLRP3 в формировании резистентности к инсулину, вызванной ожирением [16][18][20]. Исследования Esser N. и др. обнаружили различия в воспалительном профиле висцеральной жировой ткани у пациентов с метаболическими нарушениями по сравнению с пациентами без метаболических нарушений. У пациентов с метаболическими нарушениями повышается экспрессия NLRP3 и ИЛ-1β в висцеральной жировой ткани, а сама ткань инфильтрируется провоспалительными макрофагами с повышенной активностью каспазы-1 и, как следствие, увеличенным высвобождением ИЛ-1β. Высокие уровни ИЛ-1β могут способствовать нечувствительности к инсулину у людей с ожирением. Роль ИЛ-1β при хроническом воспалении, связанном с ожирением и инсулинорезистентностью, реализуется посредством двух механизмов: прямое ингибирование передачи сигналов инсулина в тканях-мишенях инсулина путем фосфорилирования серина субстрата-1 инсулинового рецептора и непрямая индукция резистентности к инсулину путем стимуляции генерации фактора некроза опухолей (ФНО-α) [25].

Инфламмасома NLRP3 может активироваться метаболическими сигнальными молекулами, такими как глюкоза, насыщенные жирные кислоты, липотоксичные церамиды, окисленные ЛПНП и холестерин, во время ожирения, что приводит к выработке ИЛ-1β и провоспалительных цитокинов [10][16][19][20]. Насыщенные жирные кислоты, такие как олеат и пальмитат, могут вызывать воспаление, опосредованное через TLR4, а потеря функции TLR4 может частично защищать от инсулинорезистентности, вызванной ожирением. В частности, связанные с ожирением повышенные уровни липотоксичных церамидов вызывают активацию каспазы-1 NLRP3-зависимым образом. Однако возможно, что в активации каспазы-1 может участвовать и ряд других индукторов [20].

## ПОТЕНЦИАЛ ПРЕПАРАТОВ, ПРИМЕНЯЕМЫХ ПРИ ТЕРАПИИ САХАРНОГО ДИАБЕТА 2-ГО ТИПА КАК ИНГИБИТОРОВ ИНФЛАММАСОМЫ NLRP3

Ряд широко применяемых препаратов для лечения СД2 продемонстрировали эффективность в качестве регуляторов активности NLRP3 [26].

В исследованиях Hill J.R. и др. оценили способность ингибировать NLRP3 у противодиабетических препаратов, производных сульфонилмочевины двух поколений. Среди всех изученных производных сульфонилмочевины глибенкламид является наиболее эффективным ингибитором NLRP3. Поскольку низкая внутриклеточная концентрация калия является одним из факторов, который совместно с сигналом от PRR вызывает активацию NLRP3, как в мышиных перитонеальных макрофагах, так и в человеческих макрофагах/моноцитах, была высказана идея, что блок K⁺ АТФ каналов может быть весьма вероятным механизмом ингибирования инфламмасомы NLRP3, опосредованным глибенкламидом [27]. Однако Lamkanf M. и др. сообщают, что блок K⁺ АТФ каналов не является обязательным условием для ингибирования инфламмасомы NLRP3. Помимо этого, препарат из группы сульфонилмочевины глипизид также ингибирует SUR 1 K⁺АТФ канала, но не влияет на активацию инфламмасомы NLRP3. Хотя точный механизм до сих пор остается неясным, глибенкламид, возможно, влияет на активацию инфламмасомы NLRP3 посредством блокады рецептора P2X7, что приводит к снижению активации каспазы-1, опосредованной инфламмасомой NLRP3, и уменьшает секрецию зрелой формы ИЛ-1β [28]. Таким образом, глибенкламид может выступать и как стимулятор секреции инсулина посредством закрытия K⁺-ATP каналов β-клеток, и как слабое противовоспалительное средство, ингибируя NLRP3 (IC50 20 мкМ) [27].

Глибенкламид выполняет защитную роль при расстройствах, связанных с воспалением, не только благодаря блокаде сигналов NLRP3 инфламмасомы/ИЛ-1 β, но и за счет не-NLRP3 механизмов, таких как сигнальные пути Sur1-Trpm4/ФНО-α и Sur1-Trpm4/Nos2/АФК. Препарат угнетает продукцию провоспалительных цитокинов и окислительный стресс, повышая активность антиоксидантных ферментов, таких как глутатионпероксидаза, супероксиддисмутаза и каталаза, и подавляет миграцию нейтрофилов и эозинофилов [29].

Метформин, представитель класса бигуанидов и препарат выбора при терапии СД2, действует через непрямую стимуляцию АМPK. Последующий запуск АМФ-зависимых внутриклеточных путей приводит к снижению активности mTOR, главного регулятора транскрипции генов и синтеза белка [30]. Активируя путь AMPK/mTOR, метформин ингибирует инфламмасому NLRP3 при диабетической кардиомиопатии [31]. Yang F. с соавт. в своих исследованиях демонстрируют, что после лечения метформином C57BL/6 мышей со стрептозотоцин-индуцированным СД уровни экспрессии mTOR, NLRP3, каспазы-1, ИЛ-1β и GSDMD-N снижаются, а назначение ингибитора АМPK приводит к отмене эффекта [32]. В исследованиях Rai R.C. и соавт. показывают, что применение метформина снижает экспрессию ASC и каспазы-1 у крыс с диет-индуцированным диабетом [33]. После двух месяцев терапии метформином у пациентов с СД2 снижается расщепление каспазы-1, активация ИЛ-1β и восстанавливается чувствительность к инсулину [16].

Пиоглитазон, селективный агониста рецептора, активируемого пероксисомными пролифераторами γ, с высоким сродством к домену связывания лиганда PPARγ описана NLRP3 ингибирующая активность, которая связана с тем, что препарат снижает высвобождение АФК и подавляет NF-κB и таким образом уменьшает повреждение клубочков при диабетической нефропатии [16][34].

Недавние исследования демонстрируют, что ингибиторы SGLT2, один из перспективных и современных классов препаратов для лечения СД2, могут ингибировать активацию воспаления, опосредованного NLRP3 на моделях ожирения, повреждения легких, инфаркта миокарда, диабетической нефропатии, депрессии и атеросклероза [35]. Согласно Benetti E. и др., эмпаглифлозин оказывает влияние на комплекс NLRP3 при моделировании ожирения и инсулинорезистентности у C57BL/6 мышей, а терапия данным препаратом не только снижает массу тела, но и контролирует уровень гликемии, при этом дозозависимо снижается активация комплекса NLRP3 и секреция ИЛ-1β [36]. Данные, полученные Liu P. и соавт., свидетельствуют о том, что эмпаглифлозин защищает ткани поджелудочной железы от повреждения при СД путем ингибирования активации инфламмасомного пути, связанного с пироптозом NLRP3/каспазы-1/GSDMD, и корректирует патологические изменения и инфильтрацию воспалительными клетками тканей поджелудочной железы db/db мышей [37]. Ye Y. и др. получены данные о том, что дапаглифлозин, помимо того, что уменьшает уровень глюкозы в тестах толерантности к глюкозе, корректирует патологические изменения в миокарде и снижает уровни мРНК NALP3, ASC, ИЛ-1β, ИЛ-6 и каспазы-1 при диабетической кардиомиопатии у мышей с СД2 [38]. Birnbaum Y. и др. демонстрируют, что использование дапаглифлозина ослабляет прогрессирование диабетической нефропатии у мышей и снижает экспрессию мРНК ASC, каспазы-1, ИЛ-6, ИЛ-1β и ФНО-α [39]. Согласно Chen H. и соавт., дапаглифлозин снижает прогрессирование диабетической кардиомиопатии и степень фиброза миокарда за счет активации AMPK/TOR пути и последующего ингибирования активации инфламмасомы NLRP3 [40].

По данным Zhu W. и соавт., применение лираглутида, синтетического аналога нативного глюкагоноподобного пептида-1, корректирует инсулинорезистентность и уменьшает морфологические проявления стеатоза печени за счет снижения экспрессии инфламмасомы NLRP3 и ИЛ-1β в печени у мышей, получавших высокожировую диету [41]. Помимо этого, лираглутид оказывает противовоспалительное и антидемиелинизирующее действие при экспериментальном аутоиммунном энцефалите у мышей, которое может быть связано с регуляцией AMPP, NLRP3 и аутофагией [42][43].

Согласно данным Birnbaum Y. и соавт., саксаглиптин предотвращает возникновение и развитие повреждения почек у мышей Akita за счет снижения активации инфламмасомы NLRP3 и провоспалительных цитокинов ФНО-α, ИЛ-1β, ИЛ-6 и ИЛ-18 [44].

Li X.X. и соавт. в своих исследованиях показывают, что акарбоза корректирует нарушения сосудистой проницаемости при СД и блокирует генерацию Nox4-зависимого супероксида, который регулирует активацию инфламмасомы NLRP3 в эндотелиальных клетках аорты крысы [45].

## АНТИ-ИЛ-1 ТЕРАПИИ ХРОНИЧЕСКОГО ВОСПАЛЕНИЯ ПРИ САХАРНОМ ДИАБЕТЕ 2-ГО ТИПА

Учитывая ключевую роль ИЛ-1β в иммуноопосредованных механизмах дисфункции β-клеток и инсулинорезистентности, анти-ИЛ-1 терапия может эффективно корректировать слабовыраженное хроническое воспаление и обосновать клиническое применение антагонистов рецептора ИЛ-1 у пациентов с СД2 [46][47].

Эффективность анти-ИЛ-1 терапии доказана как в экспериментальных исследованиях, так и в клинической практике. Анти-ИЛ-1 антитела уменьшают инфильтрацию островков и гибель β-клеток, а также улучшают секрецию инсулина и контроль глюкозы у мышей, получавших диету с высоким содержанием жиров. Кроме того, показано, что рекомбинантный антагонист рецептора ИЛ-1 анакинра частично устраняет дисфункцию β-клеток при повреждениях, вызванных глюкотоксичностью и липотоксичностью в культурах островков человека [47].

Полученные результаты об эффективности анакинры подтверждены в нескольких клинических исследованиях у пациентов с СД2. Рекомбинантный препарат антагониста рецептора ИЛ-1 улучшает секреторную функцию β-клеток, оцененную как по соотношению проинсулин/инсулин, так и в тесте толерантности к глюкозе, и снижает уровень HbA1c и провоспалительных цитокинов после 13 недель ежедневного подкожного введения. Эффект после применения препарата сохраняется в течение девяти месяцев после завершения терапии у пациентов. Однако анакинра имеет короткий период полувыведения и требует ежедневного дозирования, которое сопровождается реакцией в месте инъекции. Для повышения эффективности терапии разработаны специфические моноклональные антитела, такие как LY2189102, гевокизумаб и канакинумаб, которые позволяют сократить кратность приема препарата до одного раза в 1–3 месяца. Клинические исследования препаратов на основе моноклональных антител подтверждают эффективность антиИЛ-1 терапии при СД2. Так, еженедельное подкожное введение LY2189102 в течение 12 недель хорошо переносилось, умеренно снижало HbA1c и уровень глюкозы натощак, а также продемонстрировало значительные противовоспалительные эффекты у пациентов с СД2 [46].

Метаанализ данных восьми исследований I–IV фазы, проведенный Kataria Y. и соавт., демонстрирует, что антагонизм ИЛ-1 связан со снижением HbA1c (p<0,00001); кроме того, метарегрессионный анализ показывает значительную корреляцию между исходным СРБ и С-пептидом и снижением HbA1c [48].

Однако применение антиИЛ-1 терапии не способно эффективно устранять все патологические процессы, связанные с активацией инфламмасомы, помимо этого может увеличить риск инфекционных заболеваний [6][46][47]. Ингибирование NLRP3 низкомолекулярными соединениями, возможно, является наиболее рациональной стратегией лечения NLPR3-опосредованного хронического воспаления. По сравнению с биологическими препаратами на основе белков, низкомолекулярные ингибиторы, как правило, могут вводиться перорально и, таким образом, их применение менее инвазивно, а препараты более специфичны и экономически выгодны.

## ИНГИБИТОРЫ ИНФЛАММАСОМЫ NLRP3 В ТЕРАПИИ ДИАБЕТА 2-ГО ТИПА

При поиске структурных аналогов глибенкламида Pfizer был проведен скрининг библиотеки производных диарилсульфонилмочевины и обнаружены соединения, которые в наномолекулярных концентрациях блокируют высвобождение ИЛ-1β, одно из которых MCC950 (СР-456,773 или CRID3, рис. 3, А) ингибирует активацию NLRP3 инфламмасомы как по каноническому, так и по неканоническому пути передачи сигнала. MCC950 специфически связывается посредством высокоаффинного нековалентного взаимодействия с мотивом Walker B домена NACHT, тем самым блокируя способность NLRP3 гидролизовать АТФ и принимать или сохранять активную открытую конформацию, что ингибирует олигомеризацию NLRP3 и активацию инфламмасомы. Следует отметить, что соединение не влияет на гидролиз АТФ и не может взаимодействовать с NLRP3 в его активном состоянии [6][49].

Эффективность MCC950 оценена при моделировании различных патологических состояний, связанных с нарушением метаболизма глюкозы и инсулинорезистентностью периферических тканей. После терапии MCC950 в течение 4 месяцев снижается уровень инсулина в плазме и повышается чувствительность к инсулину у мышей с лобно-височной деменцией и нарушением гомеостаза глюкозы [50]. Терапия MCC950 улучшает тревожное и депрессивное поведение и когнитивную дисфункцию, снижает экспрессию воспалительных компонентов, связанных с NLRP3, в гиппокампе при диабетической энцефалопатии у мышей [51]. Показана эффективность MCC950 при диабетической ретинопатии. После инкубации эндотелиальных клеток сетчатки пациентов с диабетической ретинопатией, стимулированных высоким уровнем глюкозы, с MCC950 снижается апоптоз, ингибируется взаимодействие NEK7 с NLRP3 [52]. Также MCC950 уменьшает уровни ФНО-α, каспазы-1 и ИЛ-1β в гломерулярных мезангиальных клетках крыс, инкубированных с глюкозой в высокой концентрации, и корректирует признаки диабетической нефропатии in vivo [53][54]. Исследования препарата были остановлены Pfizer в связи с гепатотоксичностью, выявленной в клинических испытаниях на добровольцах.

OLT1177 (дапансутрил, рис. 3, Б) — производное β-сульфонилнитрила, селективный ингибитор инфламмасомы NLRP3, без эффектов на инфламмасомы AIM2 и NLRC4. Соединение предотвращает олигомеризацию и активацию NLRP3 путем прямого ингибирования АТФ-азной активности инфламмасомы NLRP3, что подавляет взаимодействие NLRP3 с ASC и высвобождение ИЛ-1β in vitro в PBMC человека и мышей и нейтрофилах крови человека [55]. Результаты I фазы клинических испытаний и фармакокинетических исследований демонстрируют, что OLT1177 в различных дозировках (капсулы по 100, 300 и 1000 мг): имеет длительный период полувыведения (приблизительно 23 ч), безопасен и хорошо переносится [56]. В 2024 г. Olatec Therapeutics LLC приступило к проведению многоцентрового рандомизированного двойного слепого плацебо-контролируемого исследования безопасности и эффективности перорального ингибитора NLRP3 дапансутрила у пациентов с СД2, результаты которого будут известны в 2026 г. [46].

Ряд ингибиторов NLRP3 исследуется на моделях СД и инсулинорезистентности. Так, BAY 11-7082 (рис. 3, В), производное фенилвинилсульфона, ранее описанное как необратимый ингибитор NF-kB, путем алкилирования цистеиновых структур АТФазной части NLRP3 подавляет сборку пироптосомы ASC и сигнализацию инфламмасомы NLRP3 [57]. Помимо этого, BAY ингибирует образование пор путем ковалентной модификации критического остатка цистеина C191 GSDMD [58]. BAY 11-7082 ограничивает активацию NF-kB, тем самым снижает экспрессию воспалительных цитокинов, таких как ФНО-α, ИЛ-1β, ИЛ-6, и уменьшает окислительное повреждение почек у крыс с диабетической нефропатией и ингибирует активацию NLRP3, экспрессию каспазы-1 и ИЛ-1β и пироптоз при ишемии-реперфузии у крыс со стрептозотоциновым диабетом [59][60].

CY-09 (рис. 3, Г) значительно подавляет образование инфламмасомы NLRP3 как in vivo, так и in vitro в клетках человека и мыши и является прямым и эффективным ингибитором NLRP3. Соединение напрямую взаимодействует с мотивом Walker A NLRP3 и блокирует связывание АТФ с NLRP3 и таким образом дозозависимо подавляет АТФ-, мононатрийурат- и нигерицин-индуцированную стимуляцию образования каспазы-1 и последующую секрецию ИЛ-1β, [16]. В исследованиях in vivo CY-09 повышает чувствительность к инсулину при инсулинорезистентности у мышей, получающих диету с высоким содержанием жиров [61].

NATx0 (рис. 3, Е), нитроалкеновый аналог витамина Е, ингибирует инфламмасому NLRP3 и продукцию ИЛ-1β как in vitro, так и in vivo. NATx0 блокирует транслокацию NF-kB в ядро, олигомеризацию ASC. При терапии мышей с ожирением, индуцированным диетой, соединение нормализует уровень глюкозы в тесте толерантности и увеличивает чувствительность к инсулину [62].

## ЗАКЛЮЧЕНИЕ

Роль инфламмасомы NLRP3 в системном и островковом воспалении, функции β-клеток, формировании инсулинорезистентности при СД остается темой, представляющей интерес для доклинических и клинических исследований. У широкого спектра противодиабетических препаратов продемонстрированы свойства ингибиторов NLRP3, реализуемые посредством различных сигнальных каскадов. В настоящее время в клинической практике отсутствуют препараты, способные специфически ингибировать активацию инфламмасомы NLRP3, но разработаны средства, действие которых направлено на снижение уровня ИЛ-1β, такие как анакинра, канакинумаб и гевакизумаб. Однако применение данной группы лекарственных препаратов ограничено повышением риска возникновения инфекционных заболеваний и ряда других побочных эффектов. Из прямых ингибиторов инфламасоммы NLRP3 наиболее изученными являются MCC950, OLT1177, BAY 11-7082, CY-09, однако ни один из них не одобрен для применения в клинической практике при терапии СД. Еще предстоит выяснить, может ли более целенаправленный подход к пути NLRP3 обеспечить коррекцию иммуноопосредованного воспаления при СД без побочных эффектов со стороны врожденной иммунной системы.

## ДОПОЛНИТЕЛЬНАЯ ИНФОРМАЦИЯ

Источники финансирования. Работа выполнена в рамках государственного задания Минздрава России «Хроническое воспаление, опосредованное активацией инфламмасомы NLRP3, как мишень терапии инсулинорезистентности и иммунометаболических нарушений при сахарном диабете 2-го типа», регистрационный номер 124021500036-6.

Конфликт интересов. Авторы декларируют отсутствие явных и потенциальных конфликтов интересов, связанных с содержанием настоящей статьи.

Участие авторов. Все авторы одобрили финальную версию статьи перед публикацией, выразили согласие нести ответственность за все аспекты работы, подразумевающую надлежащее изучение и решение вопросов, связанных с точностью или добросовестностью любой части работы.
